# The relationship between atrial fibrillation and NLRP3 inflammasome: a gut microbiota perspective

**DOI:** 10.3389/fimmu.2023.1273524

**Published:** 2023-11-21

**Authors:** Yaxuan Xing, Longmei Yan, Xiaoya Li, Zhijie Xu, Xianyu Wu, Huirong Gao, Yiduo Chen, Xiaojuan Ma, Jiangang Liu, Jingchun Zhang

**Affiliations:** ^1^ Xiyuan Hospital, China Academy of Chinese Medical Sciences, Beijing, China; ^2^ National Clinical Research Center for Chinese Medicine Cardiology, Xiyuan Hospital, China Academy of Chinese Medical Sciences, Beijing, China; ^3^ Graduate School, Beijing University of Chinese Medicine, Beijing, China

**Keywords:** atrial fibrillation, NLRP3 inflammasome, gut microbiota, mechanism, treatment

## Abstract

Atrial fibrillation (AF) is a common clinical arrhythmia whose pathogenesis has not been fully elucidated, and the inflammatory response plays an important role in the development of AF. The inflammasome is an important component of innate immunity and is involved in a variety of pathophysiologic processes. The NLRP3 inflammasome is by far the best studied and validated inflammasome that recognizes multiple pathogens through pattern recognition receptors of innate immunity and mediates inflammatory responses through activation of Caspase-1. Several studies have shown that NLRP3 inflammasome activation contributes to the onset and development of AF. Ecological dysregulation of the gut microbiota has been associated with the development of AF, and some evidence suggests that gut microbiota components, functional byproducts, or metabolites may induce or exacerbate the development of AF by directly or indirectly modulating the NLRP3 inflammasome. In this review, we report on the interconnection of NLRP3 inflammasomes and gut microbiota and whether this association is related to the onset and persistence of AF. We discuss the potential value of pharmacological and dietary induction in the management of AF in the context of the association between the NLRP3 inflammasome and gut microbiota. It is hoped that this review will lead to new therapeutic targets for the future management of AF.

## 1 Introduction

In clinical practice, atrial fibrillation (AF) is a relatively common arrhythmia (1), often caused by multiple risk factors, and is linked to an increased risk of mortality and morbidity associated with stroke and heart failure (2, 3). In 2010, the number of people suffering from AF was calculated to be about 33.5 million worldwide, and the incidence and prevalence have been on the rise since the 1990s, posing a great threat to human health (4). AF can be classified as paroxysmal, persistent, or permanent. The majority of patients initially experience brief, self-terminating episodes, but with the appropriate triggers and substrates, the disease will advance and become more persistent (5). The mechanics behind AF are complex and yet not fully understood. The main mechanisms include electrical remodeling and calcium remodeling of atrial myocytes, DNA repair and mitochondrial dysfunction, and inflammasome activation, et al (6). There is growing evidence that uncontrolled inflammatory signaling cause structural remodeling and electrical conduction damage in AF (7).

The NOD-like receptor protein 3(NLRP3) inflammasomes is an intracellular multiprotein complex that plays an important role in the body’s intrinsic immune response. Various foreign pathogens or intracellularly generated danger signals can be recognized by the NLRP3 inflammasome, which mediates cellular pyroptosis and causes subsequent inflammatory cell recruitment and inflammatory cascade responses that are essential for maintaining host defense functions (8). NLRP3 inflammasome-mediated inflammatory response is closely related to intestinal barrier integrity, intestinal microbial composition and intestinal metabolites (9).

The gut microbiota affects metabolism and immune regulation, and microbiota-derived molecules, whether produced by microorganisms or transformed, are major players in the dialogue with immune cells (10). Under normal conditions, the gut microbiota is a symbiosis of the organism and participates in the immune function of the intestinal internal environment and does not cause harmful immune responses. When the gut microbiota is disturbed and the intestinal barrier is malfunctioning, bacterial components and metabolites circulate throughout the body with the bloodstream, causing chronic inflammatory reactions and cascading effects.

According to the available evidence, NLRP3 inflammasomes activation is associated with the development and progression of AF, and the possible mechanisms involved are leading to increased RyR 2 protein expression and RyR 2-mediated synaptic SR Ca2+ release events as well as inducing myocardial fibrosis, and contributing to the maintenance of substrates in AF (11, 12). According to recent studies, AF is exacerbated by gut microbial imbalances, and possible causes include targeting AF substrates or inhibiting risk factors that promote the development of AF substrates (13).

The activation of NLRP3 inflammasome and the metabolic disturbance of gut microbiota both affect the process of AF, and whether there is a causal relationship between these three factors needs to be further investigated. Using a fecal microbiota transplantation (FMT) rat model, a study demonstrated that high AF susceptibility in older rats can be transmitted to younger hosts via FMT, possibly associated with a significant increase in circulating lipopolysaccharide (LPS) and glucose levels leading to upregulation of NLRP3 inflammasomes expression (14), which demonstrated that the interaction between the NLRP3 inflammasomes and the gut microbiota on the development of AF. In this review, we report the interconnection of NLRP3 inflammasomes and gut microbiota, as well as evidence linking NLRP3 inflammasomes to AF, and attempt to identify new possible pathogenic pathways from the perspective of gut microbiota-derived metabolites and whether this association is relevant to the occurrence and persistence of AF. We discuss the potential value from pharmacological and dietary induction in AF management in the context of the association between NLRP3 inflammasomes and gut microbiota. This study may provide references for new therapeutic targets of AF in the future.

## 2 NLRP3 inflammasomes

### 2.1 Composition and activation of the NLRP3 inflammasome

Inflammasomes were discovered 20 years ago as a protein complex that plays an important role in the activation of interleukin-1β (IL-1β), a key substance in inflammation, and inflammasomes are also considered to be an important component of innate immunity (15). Innate immune responses are primarily based on pattern recognition, and infectious agents share multiple molecules, called pathogen-associated molecular patterns(PAMPs) or damage-/hazard-associated molecular patterns (DAMPs), which have similar chemical properties. A small subset of pattern recognition receptors (PRRs) that recognize multiple pathogens (16, 17). Nucleotide-binding oligomerization domain NOD-like receptors (NLRs) are PRRs that recognize multiple pathogens and risk-associated products (18) Inflammasomes are multiprotein complexes assembled from PRRs following the detection of pathogenic microbes and danger signals in the host cell cytoplasm (18). To date, the best studied and validated inflammasome types are NLRP3 inflammasomes, which recognize a variety of stimuli, especially DAMPs, and are implicated in the pathogenesis of aseptic inflammatory diseases such as arthritis, atherosclerosis, et al (19–21).

**Figure 1:**
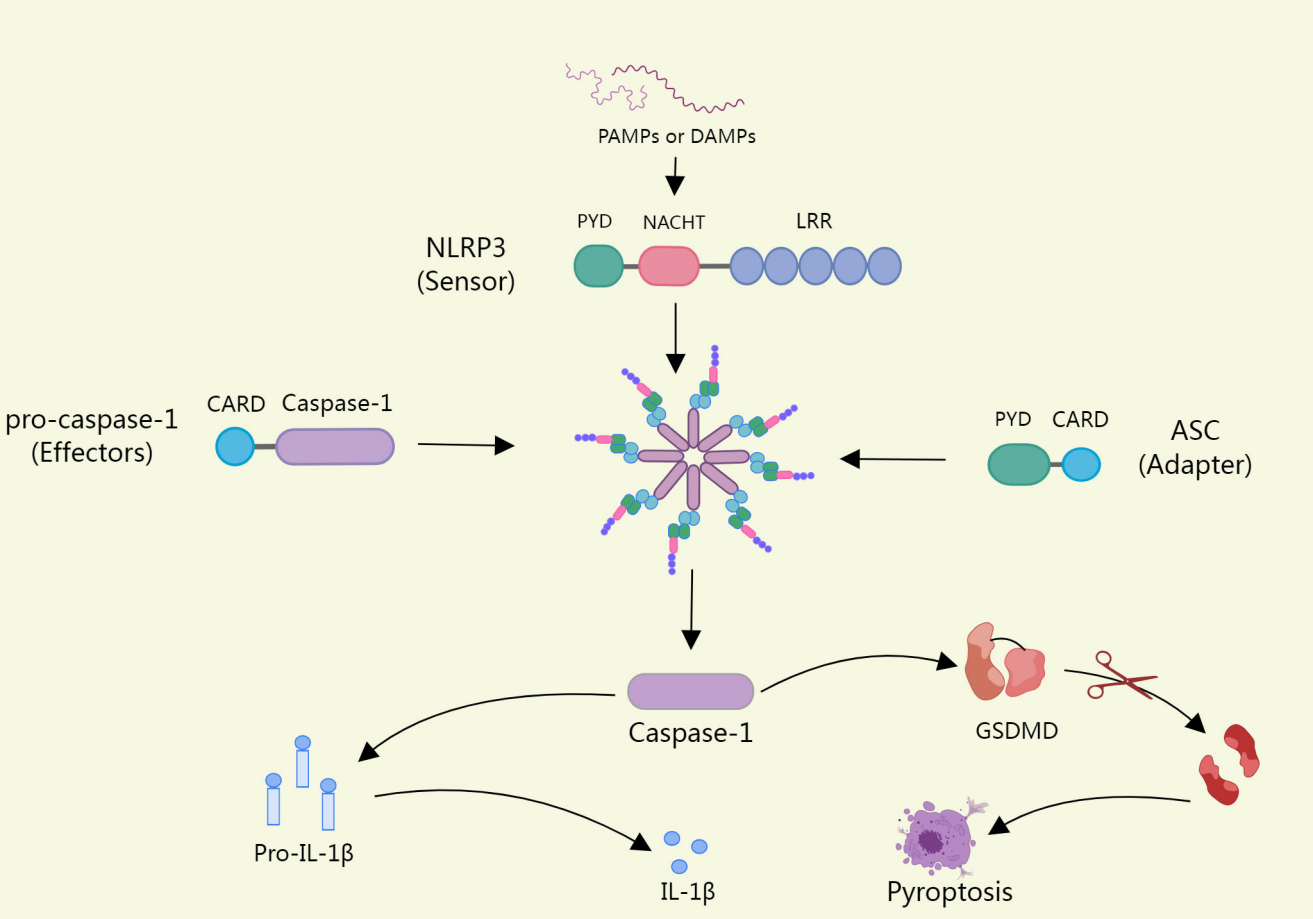
Upon pathogen stimulation, NLRP3 recruits ASCS into inflammasomes. ASC acts as a bridge linking the NLRP3 sensor to the effector Caspase-1, which assembles into intact inflammasomes, and activated caspase-1 cleaves the immature forms of Proinflammatory cytokine il-1β and Il-18, form into a mature form. In addition, Caspase-1 can also process GSDMD (Gasdermin D), release its lytic n-terminal domain (n-GSDMD), and form plasma membrane pores, which can lead to pyroptosis if a load of plasma membrane pores is high enough.

The NLRP3 inflammasome consists of a sensor (NLRP3), an adapter (ASC), and an effector(caspase-1) (22). Upon pathogen stimulation, NLRP3 is capable of forming oligomers that will recruit CARD-containing adapter protein apoptosis-associated spot-like proteins into the inflammasome via per-PYD-PYD or CARD-CARD homotypic structural domain interactions (23). ASC acts as a bridge that connects the NLRP3 sensor to the effector caspase-1 to assemble into a complete inflammasome, with activated caspase-1 cleaving immature forms of the pro-inflammatory cytokines IL-1β and interleukin-18(IL-18) to form mature forms (24). In addition, caspase-1 can also process gasdermin D(GSDMD), releasing its cleaved N-terminal structural domain (N-GSDMD) and forming a plasma membrane pore, which leads to proinflammatory necrotic cell death, also called pyroptosis, if the load on the plasma membrane pore is high enough (25). The opening of NT-GSDMD pores also promotes the release of cytokines and other inflammatory mediators (26) (**Figure 1**).

### 2.2 NLRP3 inflammasome activation pattern

Two signaling processes, priming and activation, are necessary for the NLRP3 inflammasomes to become active (27).The activation signal of the NLRP3 inflammasome is relatively unique in that most PRRs only provide a little amount of specificity for one or two closely related PAMPs or DAMPs, whereas NLRP3 can be activated by a variety of extraneous stimuli, such as viral RNA, microbial toxins and bacterial surface components, uric acid crystals, ATP, β-amyloid peptides, glycolytic products, and many other PAMPs (22).

In the typical pathway, several PAMPs or DAMPs promote NLRP3 inflammasome formation by inducing numerous molecular and cellular processes such K^+^ efflux, Cl^-^ efflux, mitochondrial dysfunction, reactive oxygen species (ROS) release, synthesis of mitochondrial DNA, and lysosomal disruption (16). In this process, most of the stimuli are activated by triggering intracellular K^+^ efflux, which in turn activates NLRP3 inflammasomes (28, 29).

**Figure 2:**
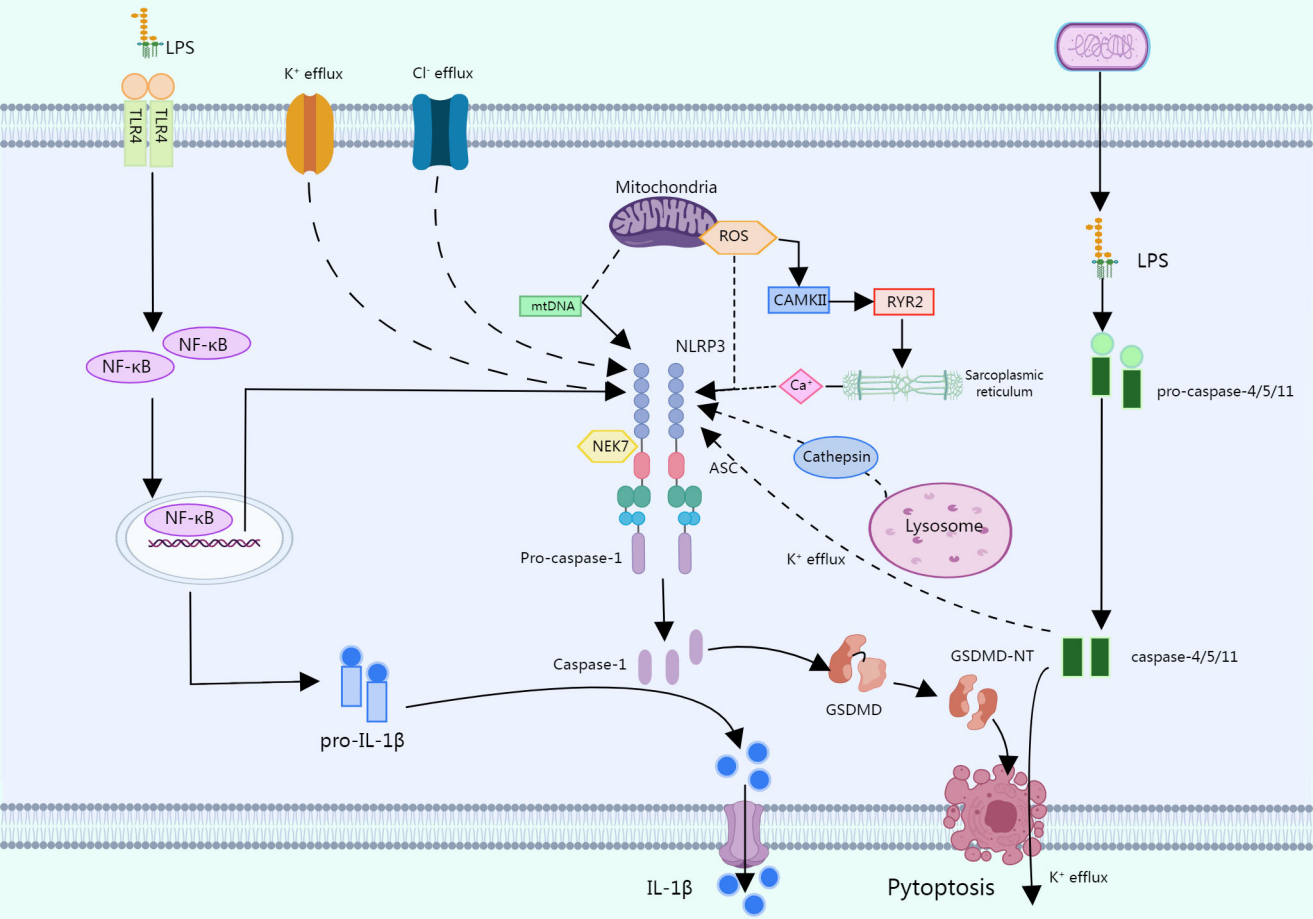
In a typical pathway, multiple PAMPs or DAMPs promote the assembly of the NLRP3 inflammasome by causing multiple molecular and cellular events such as K^+^ efflux, CI^-^ efflux, mitochondrial dysfunction, etc. LPS can activate the NLRP3 through an atypical pathway, oxidized phospholipids in LPS activate caspase-4/5 (caspase-11 in mice), and these caspases cleave GSDMD, promoting pore formation by inducing N-GSDMD insertion into the plasma membrane. The GSDMD pore allows K^+^ efflux followed by activation of the NLRP3 inflammasome, which activates caspase-1. In response to LPS, human monocytes secrete IL-1b independent of classical inflammasome stimulation but instead propagate inflammatory signals through the TLR 4-TRIF-RIPK 1-FADDCASP 8 signaling pathway upstream of NLRP3.

LPS has also been shown to activate NLRP3 inflammasomes via an atypical pathway, and oxidized phospholipids in LPS activate caspase-4/5 (caspase -11 in mice), which cleave GSDMD and promote pore formation by inducing N-GSDMD insertion into the plasma membrane. The GSDMD pore allows for K^+^ efflux and subsequent activation of the NLRP3 inflammasome, which activates caspase-1 (30, 31). Another alternative inflammatory pathway differs from the above two approaches in that in response to LPS, human monocytes secrete IL-1B independently of classical inflammasome stimulation and instead propagate inflammatory signals through the TLR4-TRIF-RIPK1-FADDCASP8 signaling pathway upstream of NLRP3 (32) (**Figure 2**).

## 3 Gut microbiota

### 3.1 Overview of gut microbiota

The human gut microbiota consists of trillions of microbial cells and thousands of bacterial species (33). A healthy gut microbiota is characterized by stability, abundance, and diversity. Firmicutes and Bacteroidetes make up 90% of the total gut microbiota. The state of the gut microbiota is considered healthy when its ratio (F/B) is low (34).

The gut microbiota is not only involved in the digestion and absorption of food in the body, but also regulates the immune system. There is a bidirectional interaction between the host innate immune system and the gut microbiota, whereby the innate immune system, upon sensing information about the metabolic state of the gut microbiota, signals the host to generate the appropriate cascade of responses, and may also modulate the composition and function of the microbiota. Inflammasome pathways are important mediators of innate immunity and can be activated by various gut microbiota and their metabolites (35). Such interactions are critical for maintaining tissue homeostasis, and perturbations between the two have emerged as key drivers of various chronic disease states (e.g., metabolic syndrome, inflammatory bowel disease, atherosclerosis, cancer, etc.) (36).

### 3.2 Derivatives and metabolites of gut microbiota

**Figure 3:**
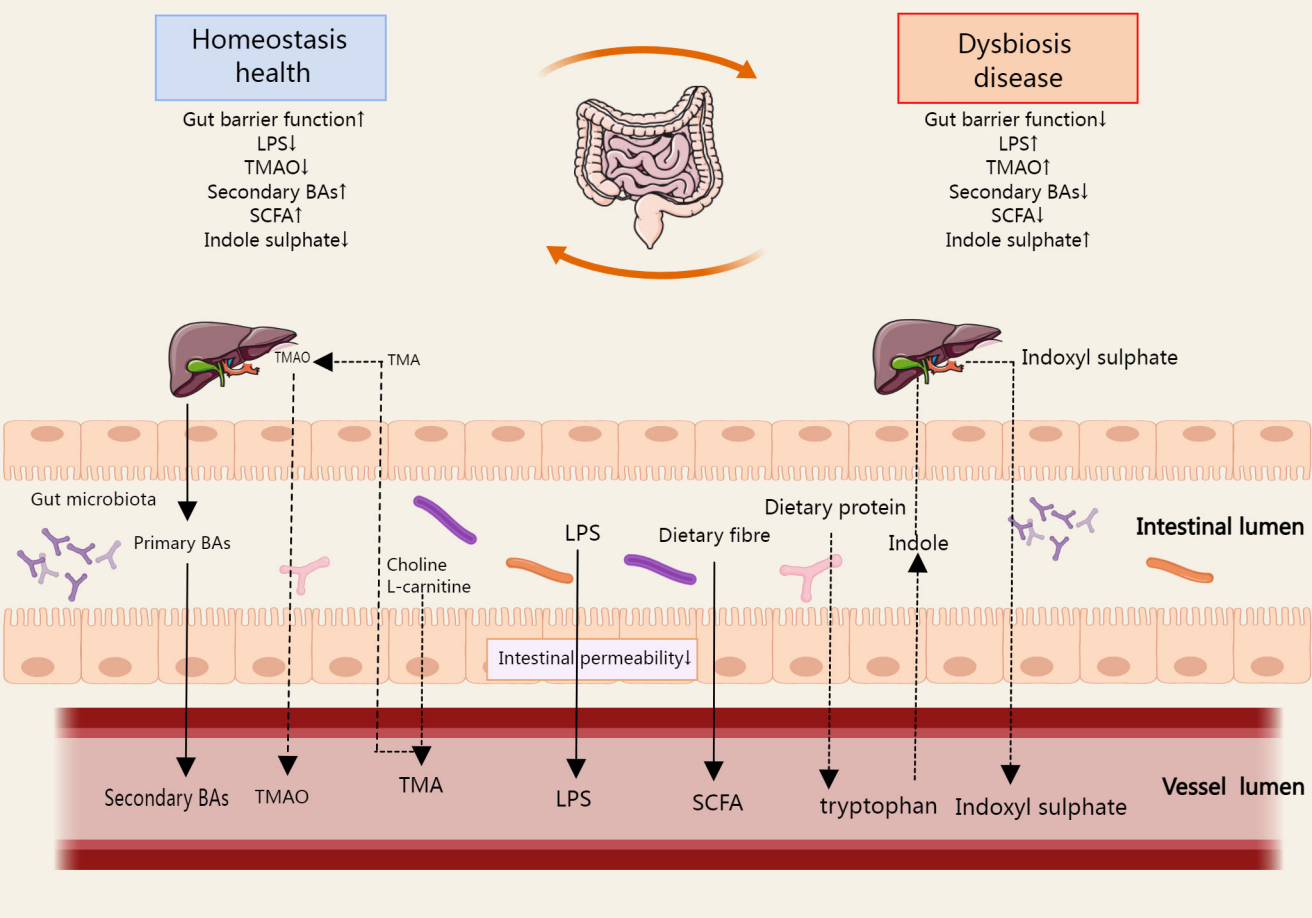
The metabolites produced during the metabolic process of gut microbial ginseng can be absorbed in the host gut, affecting the immune cell function of the gut and circulating from the gut into the host body. SCFA, TMA, Bas, and indoxyl sulfate are the typical metabolites, which affect many important metabolic pathways. In some cases, LPS enters the systemic circulation via the gut when the gut barrier function is reduced. The arrows in the figure refer to the direction of metabolism of gut microbiota derivatives and metabolites in the gut, blood circulation, and liver.

Gut microbiota derivatives and metabolites, including bacterial components, functional gut microbiota byproducts, and gut microbiota metabolites. The integrity of the intestinal epithelium prevents pathogenic invasion in the systemic circulation, thus preventing immune and inflammatory diseases (37). Lipopolysaccharide (LPS) is a major component of the outer wall of Gram-negative bacteria. It can be shed and released into the extracellular space when bacteria are disrupted or pass through outer membrane vesicles (38). In some cases, when the intestinal barrier is breached, LPS enters the human circulation through the gut and causes activation of inflammatory pathways elsewhere in the body (39). Bile acids (BAs) are synthesized by the liver and excreted with the bile into the intestine, where they are part of the digestive juices, play an important role in fat metabolism, and are typical functional by-products of the gut microbiota. The gut microbiota can promote the absorption of dietary fats and fat-soluble vitamins by influencing the type and amount of BAs in the host (40). In addition, there are many gut microbiota metabolites, typically short chain fatty acids (SCFAs), trimethylamine (TMA), and indoxyl sulfates, which affect many important metabolic pathways (**Figure 3**).

## 4 Association between NLRP3 inflammasome, gut microbiota and AF

### 4.1 NLRP3 inflammasome and AF

The onset and maintenance of AF are intimately correlated with the activation of the NLRP3 inflammasome (**Table 1**). Activation of NLRP3 Inflammasomes in atrial myocytes is one of the important mechanisms in the pathogenesis and progression of AF (11). Clinically, inflammatory indicators including IL-1 and IL-18 are linked to the development of AF and can serve as predictors of the recurrence of AF after ablation (7, 41, 42, 44, 45). Yao et al. found that NLRP3 inflammasome activity was enhanced in atrial myocytes (AMs) from patients with AF and dogs with rapid atrial pacing and that AM-restricted activation of NLRP3 in mice promoted ectopic discharge and AF-sustained substrates, thereby inducing AF (11). Enhanced activation of NLRP3-inflammasome in AMs increases caspase-1 cleavage and caspase-1-mediated apoptosis, releases inflammatory cytokines, recruits macrophages and other immune cells, and induces myocardial fibrosis and promotes the formation of AF maintenance substrates (11). NLRP3 inflammasome is hyperactivated in left atrial cells of patients with postoperative atrial fibrillation (POAF) (43), showing that the NLRP3 inflammasome may have a role in the development of AF.Metabolic disorders and cardiovascular diseases, such as obesity, diabetes, hypertension, and intestinal ecological disorders, can activate NLRP3 inflammasomes and other inflammatory cytokines in atrial cardiomyocytes, leading to atrial lesions and promoting AF (46–49). In addition, oxidative stress-induced calcium release is associated with the development of AF and activation of the NLRP3 inflammasome. Elevated levels of atrial reactive nitrogen (RNS) and reactive oxygen species (ROS) closely influence the development of AF (50, 51). Calcium ions are key molecular regulators of NLRP3 inflammasome assembly and activation (52, 53), increased intracellular ROS levels mediate the oxidation and activation of calmodulin-dependent kinase II (CaMKII), which in turn activates Ryanodine Receptor 2 (RYR2), leading to Ca^2+^ release from the sarcoplasmic reticulum (SR), thereby triggering NLRP3 inflammasome activation (54). Epicardial adipose tissue (EAT) adjacent to myocardium without fascial border (55). In the atria, EAT is associated with the incidence and severity of AF through the release of adipokines and inflammatory mediators leading to paracrine effects (44, 56–59). Related studies showed that EAT-mediated IL-1β secretion and expression were significantly higher in POAF patients than in the no-POAF group, but differences in NLRP3 inflammasome expression between groups were not confirmed in the experiments (44).

**Table 1:** Correlation studies of AF and NLRP3 inflammasome components.

Author (year)	NLRP3 inflammasome components	Experimental Methods	conclusion	References
Yao 2018	NLRP3 inflammasome	The extent of NLRP3 inflammasome activation in atrial whole-tissue lysates and cardiomyocytes from patients with paroxysmal AF or long-term persistent AF was assessed by immunoblotting. A mouse model of NLRP3-specific activation in cardiomyocytes was developed. *In vivo* electrophysiology was used to assess atrial arrhythmia vulnerability.	NLRP3-inflammasome activity was increased in atrial CMs of pAF and cAF patients. CM-KI mice developed spontaneous premature atrial contractions and inducible AF, which was attenuated by a specific NLRP3-inflammasome inhibitor, MCC950.	(11)
Ying 2010	IL-18	A case-control study design was used and 56 AF cases and 26 controls were included. AF cases were categorized as paroxysmal and persistent AF or isolated AF and AF with hypertension. Circulating levels of IL-18, tumor necrosis factor-α, and others were detected.	IL-18 is an independent factor affecting AF and is most significantly associated with all types of AF	(41)
Hua 2010	IL-1β	In a case-control study, 90 Chinese subjects were divided into 3 groups: control, paroxysmal AF, and persistent AF. After capturing serum microvesicles with a specific monoclonal antibody AD-1, the levels of IL-1β and P-selectin on the microvesicles were determined by ELISA.	Microvesicle-bound IL-1β and microvesicle-bound P-selectin levels were significantly higher in patients with persistent AF compared with normal controls.	(42)
Heijman 2020	NLRP3 inflammasome, pro-caspase-1, ASC, gasdermin-D, IL-1β	Protein expression levels of CaMKII, RyR 2, and NLRP3 inflammasome fractions in tissue homogenates or cardiomyocytes were assessed in atrial samples from 265 patients with no POAF or POAF.	CaMKII and RyR 2 protein expression was significantly increased in atrial samples from patients with POAF. patients with POAF also showed stronger expression of the activation component of the NLRP 3-inflammasome system in atrial whole-tissue homogenates and cardiomyocytes.	(43)
Serena 2022	IL-1β	EAT and atrial biopsies from 40 patients undergoing cardiac surgery were collected and screened for IL-1β and IL-1 ra in serum samples and EAT-conditioned media to evaluate atrial fibrosis histologically.	EAT-mediated IL-1β secretion and expression were significantly higher in the POAF group compared with the no-POAF group.	(44)

### 4.2 Gut microbiota and AF

The relationship between gut microbiota and AF is unclear, and recent preclinical and observational cohort studies suggest that imbalances in the composition of the gut microbiota are a factor in AF. Imbalances in the gut microbiota may contribute to obesity, hypertension, and type 2 diabetes, all of which are risk factors for the development of AF. In a small case-control study comparing 50 patients with AF to 50 matched controls, patients with AF had a disturbed microbial composition of the gut microbiota and dysregulated metabolic activity (60). Another retrospective clinical study analyzed a group of 6,763 randomly selected individuals to investigate the relationship between AF prevalence and gut microbiota. Although the gut microbiota categories did not enable differentiating between patients with prevalent or episodic AF and those without AF, the results showed a tendency to identify various genera and species. The bacterial composition shifted toward a spectrum similar to that of the hypertension and heart failure microbiomes, highlighting their common underlying pathophysiology (61). In addition, a number of derivatives and metabolite changes resulting from gut microbiota dysbiosis have also been shown to be associated with the development and exacerbation of AF (**Table 2**). Although unhealthy lifestyle, obesity, and other AF risk factors also contribute to gut microbiota dysbiosis, and the magnitude of the association between the two is influenced by many confounding factors, it may provide new insights into the mechanisms of AF and future therapeutic targets.

**Table 2:** Correlation studies of AF and gut microbiota derivatives and metabolites.

Author (year)	Gut microbiota metabolites	Experimental Methods	conclusion	References
Wang XH,et al. (2019)	Chenodeoxycholic acid	Concentrations of 12 bile acids in serum from patients with paroxysmal AF, persistent AF, type A preexcitation, and paroxysmal supraventricular tachycardia (pSVT) were determined, correlated, and analyzed in relation to areas of low voltage in the left atrium of the AF obtained by measurements of electrodissected specimens during the ablation procedure. The effect of CDCA incubation on apoptosis in mouse atrial myocytes was observed. The effect of CDCA incubation on apoptosis in mouse atrial myocytes was also observed.	Positive correlation between circulating CDCA levels and left atrial low voltage area in patients with AF. Compared with control group, CDCA (75μM,100μM) incubation with the medium significantly increased the proportion of mouse atrial myocyte apoptosis in a concentration dependent manner.	(62)
Peter P et al (2013)	BA	BA concentrations in serum samples from 250 patients were determined and analyzed for correlation with AF and ECG parameters.	Serum ursodeoxycholic acid conjugate levels were significantly lower and non-ursodeoxycholic acid levels were higher in patients with AF.	(63)
Raul S et al. (2022)	BA	The relationship between the circulating level of microbial metabolites and the incidence of CVD was systematically reviewed.	BAs were associated with all-cause mortality for AF and that elevated levels of glycopyrrolate sulfate and glycopyrrolate were associated with the risk of AF	(64)
A Alonso et al. (2015)	BA	Untargeted metabolomic analysis identified a prospective association of 118 serum metabolites with the incidence of newly diagnosed AF in 1919 African American men and women	Elevated levels of two combined BAs (glycol cholate sulfate and ethylene glycol bile sulfate) were associated with an increased incidence of AF and were not associated with other risk factors	(65)
Zhang Y,et al. (2022)	LPS	By using the FMT rat model, an attempt was made to investigate whether the heightened susceptibility to atrial fibrillation in older rats could be transmitted to younger hosts via the gut microbiota, as well as and circulating lipopolysaccharide (LPS) and glucose levels in FMT rats.	High susceptibility to atrial fibrillation in aged rats can be transmitted to young hosts via gut flora transplantation, a process associated with a dramatic increase in circulating LPS and glucose levels leading to upregulation of NLRP3 inflammasome expression	(14)
Kong B, et al. (2022)	LPS	Fecal microorganisms from normal-diet (ND) and high-fat diet (HD)-fed mice were transplanted into normal-diet mice to investigate whether they increased AF sensitivity, circulating lipopolysaccharide (LPS) levels, and TLR4/NF-κB/NLRP3 inflammatory vesicle signaling pathway expression.	Gut microbiota dysbiosis may contribute to obesity-associated AF by activating ferritinase and TLR4/NF-κB/NLRP3 inflammasome signaling pathways on atrial pathological remodeling.	(48)
Leon B et al. (2023)	LPS	Data and blood samples from patients diagnosed with atrial fibrillation for the first time (FDAF) (n = 80) and 20 controls were studied.	Circulating LPS levels were elevated in patients with new AF compared to controls and were positively associated with adverse cardiovascular events	(66)
K Aoki et al. (2015)	IS	IS and AF susceptibility as well as oxidative stress and inflammation levels *in vivo* were observed in 5/6 nephrectomized mice. In cultured atrial fibroblasts incubated with IS, upregulation around the expression of markers of oxidative stress, inflammation and prefibrotic factors was observed.	IS promotes atrial fibrosis and AF and has a direct effect on the progression of AF substrates.	(67)
Zuo K,et al. (2022)	SCFA	A 48-person cross-sectional study based on GC-MS metabolomics was conducted to explore the relationship between fecal SCFA levels and AF characteristics. Mouse models fed diets deficient or rich in dietary fiber were established to elucidate the pathophysiological role of SCFA in AF susceptibility, atrial remodeling, and G protein-coupled receptor 43 (GPR43)/NLRP3 signaling.	Significantly lower SCFA levels in feces of AF patients. In animal experiments, supplementation with SCFA prevented upregulation of CAMKII and Ryr2 phosphorylation, disorderly fibrosis, collagen expression, and inflammasome activation in atrial tissue.	(68)
Lilei Yu,et al (2019)	TMAO	TMAO or saline was locally injected into the 4 major atrial ganglion plexuses (GP) in normal dogs to observe its effect on cardiac ANS and AF induction. In the rapid atrial pacing (RAP)-induced AF model, TMAO or saline was injected into the 4 major atrial GPs to study its effect on AF progression.	TMAO exacerbates the electrophysiological instability of normal canine atria, and the increase in inflammatory cytokines induced by activation of the p65 NF-κB signaling pathway in the plexus may be one of its mechanisms of action.	(69)
S Lai et al. (2018)	TMAO	A metabolomics study of heart appendage and plasma samples from 165 patients with CVD demonstrated comparative differences.	Higher levels of choline in atrial appendage and plasma samples from AF patients compared to the general population of subjects.	(70)
G F T Svingen et al. (2018)	TMAO	The relationship between plasma TMAO and atrial fibrillation (AF) was explored in two cohort studies.	Plasma TMAO was associated with incident AF and this relationship was independent of traditional AF risk factors and dietary choline intake.	(71)

### 4.3 The relevance of the three factors

**Figure 4:**
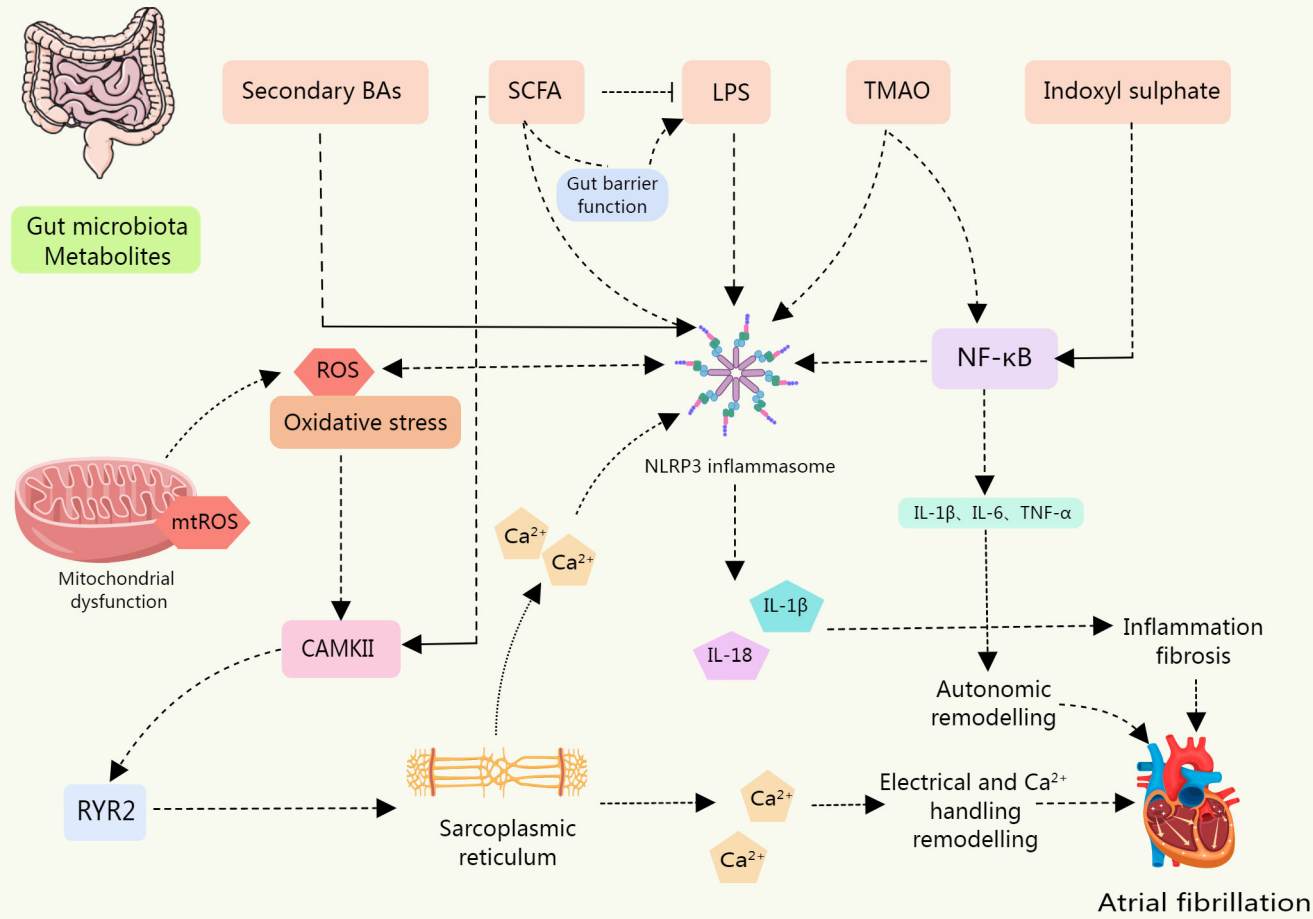
The levels of NLRP3 inflammasome, IL-1β, and IL-18 were upregulated in IS-treated cardiomyocytes, leading to cardiomyocyte apoptosis; BAs are a class of DAMPs that activate the NLRP3 inflammasome in a calcium influx dependent manner; LPS is involved in the typical and atypical activation of NLRP3 inflammasome. The sharp increase in circulating LPS level is associated with the up-regulation of NLRP3 inflammasome expression in rats, which increases their susceptibility to AF. SCFA is a potential contributor to the pathogenesis of AF. SCFAs promote the improvement of intestinal barrier function. Low SCFA levels lead to decreased intestinal integrity and promote the entry of substances such as LPS into blood and tissues. SCFA supplementation can prevent the up-regulation of phosphorylation of CAMK II and RyR2, and prevent disordered fibrosis, collagen expression, and NLRP3 inflammasome activation in atrial tissue. TMAO may be involved in the activation of the p65 NF-κB signaling pathway by activating NLRP3. Metabolites of gut microbiota can induce inflammation, myocardial fibrosis, autonomic remodeling, electrical remodeling, and other pathological processes of AF by directly or indirectly activating NLRP3 inflammasome.

The association between the onset and progression of AF and the NLRP3 inflammasome is direct. The activation of NLRP3 inflammasomes can induce or exacerbate AF. Gut microbiota metabolites and derivatives differentially modulate the NLRP3 inflammasome (**Table 3**). And this section will explore the links that exist between gut microbiota derivatives or metabolites and NLRP3 inflammasomes and AF in the context of both direct and indirect evidence in this regard (**Figure 4**).

**Table 3:** Modulation of the NLRP3 inflammasome by gut microbial derivatives and metabolites.

Derivatives and metabolites of gut microbiota	Modulation of the NLRP3 inflammasome
LPS	Involved in the typical and atypical activation processes of the NLRP3 inflammasome, promoting the release of IL-1β and IL-18.
BAs	BAs are a class of DAMPs that activate NLRP3 inflammasomes in a Ca2+ influx-dependent manner, whereas FXR inhibits NLRP3 inflammasome activity through physical interaction with NLRP3 and caspase-1.
IS	Related studies have confirmed the ability of IS to promote cardiomyocyte apoptosis and fibrosis by upregulating NLRP3 inflammatory vesicle components (NLRP3, ASC, and protease-1).
TMAO	TMAO is associated with the formation and activation of NLRP3 inflammasome. ROS production is the most common pathway in inflammasome assembly. A related study found that TMAO could activate NLRP3 inflammatory vesicles by inhibiting the SIRT3-SOD2-mitochondrial ROS signaling pathway.
SCFA	SCFAs are capable of inducing K+ efflux of the epithelial cell membrane in a GPR43-dependent manner, which has been linked to the activation of the NLRP3 inflammasome. SCFA also contributes significantly to the function of the intestinal barrier, and low levels of SCFAs can lead to a decrease in the engagement of metabolite-sensing G protein-coupled receptors, which can compromise the integrity of the gut and facilitate the entry of substances, such as LPS, into the bloodstream and tissues, which can contribute to the activation of the NLRP3 inflammasome.

#### 4.3.1 Effect of NLRP3 inflammasome and bacterial components on AF

LPS, a chemical component unique to the outer wall layer of Gram-negative bacteria, can induce inflammatory expression through activation of the receptor Toll-like receptor 4 (TLR4), eliciting a cascade response of immune stimulation and toxic pathophysiological activity in the body (72). LPS is involved in the typical and atypical activation processes of the NLRP3 inflammasome, promoting the release of IL-1β and IL-18, which are the main pathways of inflammation (73). Increased intestinal permeability contributes to the translocation of LPS on the intestinal epithelium, which leads to a moderate increase in blood LPS levels and causes systemic inflammation, defined as metabolic endotoxemia (74). LPS-induced metabolic endotoxemia is associated with obesity and increased cardiovascular risk and insulin resistance (75).

Recent evidence suggests that LPS is associated with the pathogenesis of AF. Animal experiments have shown that LPS increases the expression levels of inflammatory cytokines and L-type calcium channel proteins, shortens the effective atrial expiration period (ERP), and thus promotes the development of AF (11, 76). Zhang et al. demonstrated by using a FMT rat model that the high susceptibility of aged rats to AF can be transmitted to younger hosts, a process associated with a dramatic increase in circulating LPS and glucose levels leading to an upregulation of NLRP3 inflammasome expression (14). Kong et al. found that transplantation of fecal microorganisms derived from high-fat diet-fed mice into normal diet mice resulted in significantly increased susceptibility to AF, significantly higher circulating LPS levels, enhanced expression of intracardial ferritin, and enhanced activation of the TLR4/NF-κB/NLRP3 inflammasome signaling pathway relative to controls. Significant improvement in atrial fibrosis and reduction in susceptibility to AF induced by obesity-related gut ecological dysregulation through inhibition of ferritin excess or NLRP3 inflammasome signaling pathways (48).

LPS does not appear to directly cause new-onset AF, and in a study in which 652 healthy men (all without a history of AF) were infused with LPS and underwent continuous cardiac monitoring to assess rhythm, experimental LPS excitation resulted in a significant increase in acute inflammation-related indices but did not increase the propensity for new-onset AF in young, low-risk individuals. A similar conclusion was obtained in an animal experiment in which an experimental autoimmune myocarditis model was established and AF was induced by atrial electrical stimulation, and AF was induced in the chronic phase of myocarditis rats, whereas AF did not occur in the acute phase of myocarditis or LPS-infused rats (77). It may suggests that acute inflammation per se does not increase the incidence of AF induction, and that atrial structural remodeling due to inflammation and hemodynamic effects is necessary to induce AF. A study showed that circulating LPS levels were elevated in patients with new AF compared to controls and were positively associated with adverse cardiovascular events, suggesting that improving the intestinal barrier and reducing endotoxemia may be a potential target for treating cardiovascular diseases(CVD) and preventing complications (66).

#### 4.3.2 Effect of NLRP3 inflammasome and functional gut microbiota byproducts on AF

An essential part of bile, BAs is crucial for the metabolism of glucolipids and the release of stored energy (78). BAs are produced in the liver and kept in the gallbladder. After eating, BAs are secreted into the intestine and metabolized by gut microorganisms. A total of 5% of BAs are expelled in feces, while 95% of BAs are reabsorbed in the ileum and transported back to the liver through enterohepatic circulation (79). Through gut flora transformation, BAs regulate multiple metabolic pathways in the host through nuclear farnesoid X receptor (FXR) and G protein-coupled membrane receptor (GPCR)-mediated signaling. Also, by triggering the innate immune response, BAs may have a direct or indirect impact on the composition of the gut’s microbiota (80). Primary BA (e.g. goose deoxycholic acid) is formed by combining with amino acids (e.g. taurine or glycine) to form bile salts, which are then further secreted into the intestine. The primary BA is further converted by the gut microbiota to form secondary BA (e.g. ursodeoxycholic acid). Dysbiosis of the gut microbiota regulates the BA ratio, decreasing the concentration of secondary BA and increasing the concentration of primary BA (13).

BA is a metabolite that circulates and binds to its receptor expressed in multiple tissues, cells, and organs throughout the body. BAs serve as crucial mediators of intestinal microecology and NLRP3 inflammasome activation (81). There is growing evidence that activation of the NLRP3 inflammasome by BAs is a major feature of systemic chronic low-grade inflammation. BAs are a class of DAMPs that activate NLRP3 inflammasomes in a Ca^2+^ influx-dependent manner, whereas FXR inhibits NLRP3 inflammasome activity through physical interaction with NLRP3 and caspase-1 (82). It has been found that BAs have different regulatory effects on NLRP3 in different inflammatory states. Under non-inflammatory conditions, supplementation with BAs activated the NLRP3 inflammasome in THP-1 differentiated macrophages and promoted inflammation. However, in LPS-induced inflammatory macrophages, BAs inhibited NLRP3 inflammasomes and attenuated inflammation (83).

A Study has shown that serum levels of ursodeoxycholic acid are significantly lower and non-ursodeoxycholic acid levels are higher in patients with AF (63). Another study measured serum concentrations of 12 bile acids separately in patients with different types of AF and found that circulating levels of goose deoxycholic acid (CDCA) were elevated in AF patients. CDCA caused a dose-dependent increase in atrial myocyte apoptosis in mice, suggesting that CDCA may play an important role in the structural remodeling process of AF (62). A systematic review of gut microbial-derived metabolites showed that BAs were associated with all-cause mortality for AF and that elevated levels of glycopyrrolate sulfate and glycopyrrolate were associated with the risk of AF (64). A prospective cohort study based on 1919 African Americans showed that increased levels of two combined BAs (ethylene glycol cholate sulfate and ethylene glycol cholate sulfate) were associated with increased incidence of AF and were independent of other risk factors (65).

The function of BA in immune system regulation has been validated. The regulatory effects of BA on the NLRP3 inflammasome differ across various states (81). While existing studies have demonstrated a link between AF and BA metabolism to a certain extent, and have characterized serum BA levels in AF patients, the specific mechanisms underlying how BA metabolism affects AF remain less well-studied. No research has explored the association between AF and BAs from inflammatory signaling pathways, indicating a potential area for future investigation.

#### 4.3.3 Effect of NLRP3 inflammasome and gut microbiota metabolites on AF

Indoxyl sulfate (IS), a specific product of the gut microbiota metabolizing dietary tryptophan (from protein foods), is one of the most common uremic toxins. As dietary tryptophan reaches the colon, it is transformed into indole by microbial action and absorbed into the bloodstream. From there, the indole is further metabolized by the liver to produce IS and finally excreted by the kidneys (84). CKD is associated with a higher incidence of AF, but the mechanism is unclear (85). An animal study showed that IS increases the occurrence of pulmonary venous and atrial arrhythmias through oxidative stress, which may be associated with the occurrence of AF in CKD patients (86). Another animal experiment showed that mice with higher levels of IS, *in vivo* oxidative stress and inflammation after 5/6 nephrectomy were more prone to induce AF, while nephrectomy-induced changes were attenuated after AST-120 (an adsorbent for uremic toxins) reduced IS circulating levels (67). There is no direct evidence that IS affects AF through activation of the NLRP3 inflammasome, but there is correlative evidence confirming the ability of IS to upregulate NLRP3 inflammasome components (NLRP3, ASC, and procaspase-1), which contributes to cardiomyocyte apoptosis and fibrosis. *In vitro* treatment of mouse cardiomyocytes with IS resulted in upregulation of NLRP3 inflammasome, IL-1β and IL-18 levels in IS-treated cardiomyocytes through activation of the NF-κB signaling pathway, leading to apoptosis (87). To avoid AF brought on by renal insufficiency, IS may be a prospective therapeutic target and a risk factor for AF in renal failure.

The gut microbiota is engaged in the fermentation of dietary fiber and glucose to create SCFA, such as acetate, butyrate, and propionate, which are vital for preserving the *in vivo* homeostasis of the gut microbiota and host immunity. SCFAs can directly activate G-coupled receptors and inhibit histone deacetylases, which can repair gut barrier dysfunction and affect various physiological processes (88, 89). Related studies have shown that fiber can promote the release of SCFAs through intestinal microbial fermentation, that SCFAs can induce epithelial cell membrane K^+^ efflux in a GPR43-dependent manner, that this mechanism is associated with NLRP3 activation, and that IL-1β release following inflammasome activation contributes to intestinal internal environmental stability (90). SCFAs might have a role in the pathophysiology of AF. SCFAs contribute to intestinal barrier function, and low SCFA levels lead to decreased metabolite sensing G protein-coupled receptor engagement, which compromises intestinal integrity and promotes the entry of substances such as LPS into blood and tissues (91). A metabolomics-based cross-sectional study showed significantly lower fecal SCFA levels in AF patients (68). Animal experiments have shown that dietary fiber deficiency in mice increases susceptibility to AF during pacing, and supplementation with SCFAs may have a protective effect. Supplementation with SCFAs prevents upregulation of CAMKII and RyR2 phosphorylation and prevents disordered fibrosis, collagen expression and NLRP3 inflammasome activation in atrial tissue (68).

Choline, L-carnitine, betaine, and other choline-containing substances are the major dietary precursors of trimethylamine oxide (TMAO), which is a significant gut microbe-dependent metabolite (92). The gut microbiota is a key factor in the production of TMAO and can metabolize nutrient precursors from the diet into TMA (93, 94). TMA is absorbed through the intestine and transported through the circulatory system to the liver, where it is processed by hepatic flavin monooxygenase (FMO) to form TMAO (95, 96). In mice, the addition of TMAO, carnitine or choline to the diet alters the microbial composition of the cecum (93). In metabolic diseases, with increased dietary choline and L-carnitine intake, the microbiota is altered, resulting in elevated plasma TMAO levels (97). TMAO is associated with the formation and activation of NLRP3 inflammasomes and may be an important initiating mechanism for turning on the endothelial inflammatory response leading to endothelial dysfunction (98). A related study found that TMAO can activate NLRP3 inflammasome and promote vascular inflammation by inhibiting SIRT3-SOD2-mitochondrial ROS signaling pathway (99). ROS generation is the most common pathway in inflammasome assembly (100). In contrast, the SIRT3-SOD2 linkage pathway deacetylates mitochondrial proteins and limits the accumulation of mitochondrial ROS (101).

Elevated TMAO concentrations may be associated with diet, changes in the composition of the gut microbiota, intestinal dysbiosis, or impaired gut-blood barrier, and can increase the risk of cardiovascular disease, and metabolic syndrome (102, 103). These are risk factors that promote AF and can increase susceptibility to AF. By locally injecting TMAO or saline into the four major atrial ganglion plexuses (GPs) of normal dogs or dogs in an AF-induced model, Yu et al. found that TMAO increased atrial electrophysiological instability in normal dogs and exacerbated acute electrical remodeling in the AF model by promoting autonomic remodeling. These alterations may be associated with the activation of the p65 NF-κB signaling pathway and increased inflammatory cytokines in GPs (69). Cold exposure is an important risk factor for AF (104). Cold exposure increased the susceptibility of rats to AF and also led to a decrease in the abundance of A. muciniphila in the rat intestine, which increased the level of TMAO by modulating changes in microbial enzymes. Similarly, human participants’ plasma TMAO levels increased over time as the temperature dropped. Elevated TMAO enhanced infiltration of M1 macrophages in the atria, increased the expression of Casp1-p20 and cleaved GSDMD, induced apoptosis, and ultimately led to structural remodeling of the atria. However, mice with conditional deletion of caspase1 exhibit resistance to cold-related AF (105). These are components associated with the NLRP3 inflammasome, but NLRP3 inflammasome expression was not further investigated in this study. Direct mechanistic studies related to TMAO, AF, and NLRP3 inflammasomes are almost nonexistent, and this is a new area worth exploring. In addition, a metabolomic study of atrial appendage samples and plasma samples from 165 patients with cardiovascular disease (CVD) revealed higher levels of choline in atrial appendage and plasma samples from AF patients compared to the general population of subjects (70). The results of two cohort studies showed that plasma TMAO was positively associated with long-term AF events, a prospective relationship independent of traditional AF risk factors and potential confounders includes dietary intake of TMAO precursors (e.g., choline and betaine) (71).

## 5 Treatment strategies for AF

### 5.1 Inhibition of NLRP3 inflammasome activation

Targeting inflammatory signaling has become an emerging option for AF management. At this stage, NLRP 3 inhibitors, caspase-1 inhibitors, and IL-1β antagonists have been developed successively (106). Regulation of NLRP3 inflammasome itself or its activation mechanism can inhibit the release of inflammatory factors and apoptosis, thereby alleviating myocardial fibrosis. Inhibition of the NLRP3 inflammatory pathway also affects gut Microbiota (107).

The diaryl sulfonylurea compound MCC950 is a specific inhibitor of NLRP3 and is generally believed to act on the assembly portion of the NLRP3 inflammasome. K^+^ efflux and the interaction of NEK7 with NLRP3 are considered to be potential targets of MCC950 (108). In the FMT rat model, significant elevation of LPS and glucose levels led to upregulation of NLRP3 inflammasome expression, which promoted the development of AF, and inhibition of NLRP3 inflammasome with MCC950 led to reduced susceptibility to AF (14).

Canakinumab is a monoclonal antibody that blocks IL-1β-mediated inflammatory pathways (109). A randomized controlled study of patients with persistent AF showed that anti-inflammatory treatment with canakinumab after electrical cardioversion did not reduce AF recurrence (110), but promising trends were seen in this experiment. Canakinumab reduced major cardiac events in patients with atherosclerosis in the CANTOS trial (Canakinumab Anti-Inflammatory Thrombosis Outcomes Study) (111).

Colchicine is a microtubule-destroying drug that was first used in the treatment of gout and has a variety of anti-inflammatory effects. The anti-inflammatory mechanism of colchicine is unclear, but there is evidence that it reduces inflammasome-mediated IL-1β and IL-18 production by inhibiting the assembly and activation of the NLRP3 inflammasome (112). ACC/AHA guidelines suggest that colchicine may be considered for the prevention of POAF (Class IIb evidence level B) (113). Some current clinical evidence evaluates the effect of colchicine on cardiovascular diseases such as pericarditis, postoperative and post-ablation AF, and coronary artery disease (114–116). Colchicine has been tested in several clinical trials for the treatment of POAF (117–119). However, the designs of these studies differed in terms of a drug loading dose, AF treatment modality, time to treatment initiation (pre/postoperative), and duration of follow-up. Therefore, additional trials are needed to demonstrate the potential value of colchicine in the treatment of different forms of AF.

### 5.2 Diet modification

Short-and long-term changes in gut microbiota are closely related to diet. Diets rich in animal fats and saturated fats may alter the intestinal flora by increasing LPS, increasing TMAO, and decreasing SCFA (120) Altered intestinal permeability to LPS may be the trigger of low-grade systemic inflammation. Cani et al. found that a high-fat diet increased the proportion of LPS-containing microbiota in the mouse gut and dysregulated inflammatory tone, triggering weight gain and insulin resistance (74). High-fiber diets are associated with increased SCFA production in the gut, and when dietary fiber is in short supply, not only is SCFA production reduced, but it also results in the gut microbiota using less favorable substrates, such as amino acids and host mucins, for energy (88, 121, 122). In addition, the type of diet can affect microbial-derived TMA and may influence TMAO levels. Compared to vegans/vegetarians, omnivorous human subjects consuming L-carnitine produced more TMAO (123).

The Mediterranean diet is plant-based, rich in fiber and omega-3 fatty acids, and low in animal protein and saturated fat. Ghosh et al. analyzed the gut microbiota before and after a 12-month MedDiet intervention (NU-AGE diet) for elderly subjects in 612 non-frail or prematurely aged subjects from five European countries (UK, France, Netherlands, Italy, and Poland) and found that adherence to the Mediterranean diet modulated microbiota changes associated with increased production of short/branched-chain fatty acids and decreased production of secondary BAs, p-cresol, ethanol and carbon dioxide (124). A study investigating the dietary habits of AF patients found that AF patients were less likely to report adherence to a Mediterranean diet, a lower intake of plant foods such as nuts, vegetables, and fruits, and a preference for white meat over red meat (125), but the sample size included in that trial was small and retrospective and larger clinical studies are needed to confirm the extent to which high-quality dietary patterns such as the Mediterranean diet affect the onset of AF.

However, there is still a lack of studies based on dietary habits, gut microbiota, and the development of AF, and whether dietary habits can affect AF by influencing changes in the gut microbiota and its metabolites remains a hot topic for further research.

### 5.3 Gut microbial reconstruction

There is no clear evidence whether targeted modulation of gut microecology, including administration of probiotics, administration of prebiotics, or gut flora transplantation, can influence AF progression. Probiotics mainly include Lactobacillus spp. and Bifidobacterium spp. and have the functions of enhancing the barrier function of the gastrointestinal tract, inhibiting the growth of pathogens, and suppressing their harmful toxins (126). Probiotics can mitigate AF-related risk factors (obesity, abnormal lipid metabolism, inflammation, etc.) (127).

Prebiotics are not digested and absorbed by the host, but can selectively promote the metabolism and proliferation of beneficial bacteria in the body, thus improving host health. An animal study found that resveratrol attenuated TMAO-induced atherosclerosis by reducing TMAO levels and through gut microbiota remodeling (128). Oligogalactose increases SCFA and reduces high-fat diet-induced LPS production (129).

FMT is an intervention to restore a patient’s intestinal flora by transferring a specially prepared stool sample from a healthy donor to a recipient (130). Reduced atrial NLRP3 inflammasome activity and decreased atrial fibrosis in aged rats by transferring intestinal flora from juvenile to aged rats (14). In another study, the transplantation of cecum contents from normotensive rats into spontaneously hypertensive rats lowered blood pressure, while normotensive rats had increased blood pressure after FMT in spontaneously hypertensive rats (131). FMT may be a potential pathway and research hotspot for the treatment of CVD in the future. The exact effect of FMT on the AF matrix and its mechanism still need more investigation.

## 6 Conclusion and outlook

Activation of the NLRP3 inflammasome and dysregulation of intestinal microecology is a potential target factor that may provide new therapeutic avenues for AF. The interactions between NLRP3 inflammasome and intestinal microecological metabolic processes have been gradually elucidated, both of which affect the developmental process of AF, and whether there is a synergistic or causal relationship needs the further investigation. Gut microbial dysbiosis may promote increased AF susceptibility or AF substrates by influencing their derivatives and metabolites to modulate oxidative stress responses, regulate metabolism, and upregulate NLRP3 inflammasome expression, leading to cardiac inflammatory responses, myocardial fibrosis, or metabolic disturbances. In this review, less high-quality direct evidence demonstrated a relationship between the gut microbiota-AF-NLRP3 inflammasome axis. Only SCFA and LPS have been shown to be directly associated with the NLRP3 inflammasome and AF. TMAO, BAs and IS although all shown to be associated with NLRP3 inflammasome activation and AF, respectively, have not yet been found to be directly associated with all three. This review collates existing studies on gut microbiota derivatives and metabolites and immune response and AF occurrence, with a view to providing a reference for in-depth research in this area.

More valuable researches should inspire the development of new theoretical and drug targets in AF. This article may provide insights into the use of pharmacological inhibition of NLRP3 inflammasome activation, dietary modification, and re-establishment of gut microbiota for the clinical management of AF and its comorbidities. However, most studies used single-factor intervention analysis in this manuscript, which cannot better explain the relationship between the three. Therefore, it is urgent to explore the specific regulatory mechanism of NLRP3 inflammasome and intestinal microecology of AF patients through more comprehensive researches.

## Author contributions

YX: Writing – original draft, Writing – review & editing. LY: Writing – review & editing. XL: Writing – review & editing. ZX: Writing – review & editing. XW: Writing – review & editing. HG: Writing – review & editing. YC: Writing – review & editing. XM: Writing – original draft, Writing – review & editing. JL: Writing – original draft, Writing – review & editing. JZ: Writing – original draft, Writing – review & editing.
